# The genus *Microserangium* Miyatake (Coleoptera, Coccinellidae) from China

**DOI:** 10.3897/zookeys.359.6057

**Published:** 2013-12-05

**Authors:** Xingmin Wang, Adam Ślipiński, Shunxiang Ren

**Affiliations:** 1 Engineering Research Center of Biological Control, Ministry of Education, South China Agricultural University, Guangzhou, 510642 China; 2 CSIRO Ecosystem Sciences, Australian National Insect Collection, GPO Box 1700, Canberra, ACT 2601, Australia

**Keywords:** Coleoptera, Coccinellidae, Serangiini, *Microserangium*, new species, China

## Abstract

The genus *Microserangium* Chapinfrom China is reviewed. Nine species are recognized, including seven new species: *M. erythrinum* Wang & Ren, **sp. n.**, *M. fuscum* Wang & Ren, **sp. n.**, *M. glossoides* Wang & Ren, **sp. n.**, *M. shennongensis* Wang & Ren, **sp. n.**, *M. semilunatum* Wang & Ren, **sp. n.**, *M. deltoides* Wang & Ren, **sp. n.**, *M. dactylicum* Wang & Ren, **sp. n.** Male genitalia of *M. hainanensis* Miyatake, 1961 are described for the first time. All species are described and illustrated. A key and distribution map to the known species from China are given.

## Introduction

The genus *Microserangium* was established by Miyatake (1961a) with *Microserangium shikokense* Miyatake, 1961 as the type species, based on its antenna composed of nine antennomeres with the third antennomere strongly triangular, roughly quadrate mandible, and tarsi with 3 tarsomeres. *Microserangium* is a small genus of Serangiini, mainly reported in the Oriental region (Miyatake 1994).

Chapin (1940) proposed the new genus *Serangiella*, and characterized it as possessing nine antennomeres with the third strongly triangular, a roughly quadrate mandible, and tarsi composed of 4 tarsomeres. He designated *Oeneis flavescens* Motschulsky, 1866, as the type species of *Serangiella*, but without seeing its type material. Ślipiński and Burckhardt (2006) examined the specimens used by Chapin (1940) for the description of *Serangiella* and found that tarsomeres 3 and 4 are partially fused in some specimens. They concluded that this character is unreliable for separating genera of the tribe Serangiini,and synonymised *Microserangium* with *Serangiella*.

However, Hoàng (1977) found that the type species of *Serangiella* was misidentified, and *Oeneis flavescens* Motschulsky did not belong to Coccinellidae. He elected to consider Chapin’s misidentified species as a new binomen, *Serangiella flavescens* Chapin, 1940, and treated it as type species of *Serangiella*. Unfortunately, this action was not in accord with article 70 of the 1964 ICZN, which required that cases of misidentified type species be submitted to the Commission for ruling. It was not until the 1999 edition (article 70.3) that authors were given the option of solving these issues themselves by designating as the type species either the species originally cited, or the species actually involved in the misidentification. Ślipiński and Burckhardt (2006) attempted to fix Hoang’s type designation by citing article 70.3 along with the name previously cited as type species (*Oeneis flavescens* Motschulsky) and the name of the species selected (*Serangiella flavescens* Chapin). This would have achieved their goal, except that *Serangiella flavescens* Chapin is an unavailable name due to the fact the species was never formally described. Chapin cannot be considered to have described this species even though his genus *Serangiella* is clearly based upon it, and neither Hoang (1977) nor Ślipiński and Burckhardt (2006) provided an official description of the new species in a manner that would satisfy the respective versions of the ICZN in effect during the time of their publications. Therefore, we consider *Serangiella* as an unavailable name and restore *Microserangium* as the valid name for this genus.

At present, *Microserangium* has eight species from the Old World, mostly from Asia with *Microserangium okinawense* Miyatake and *Microserangium hainanensis* Miyatake recorded from China (Miyatake 1961a, 1961b, 1994, Pang et al. 2004, Ślipiński and Burckhardt 2006, Ren et al. 2009, Escalona and Ślipiński 2012, Wang and Ren 2012). In the present paper, nine species of *Microserangium* are reported from China, including seven new species.

## Materials and methods

All recently collected specimens from China were preserved in 85% ethanol. External morphology was observed with a dissecting stereoscope (SteREO Discovery V20, Zeiss). The following measurements were made with an ocular micrometer: total length, from apical margin of clypeus to apex of elytra (TL); Total width, across both elytra at widest part (TW=EW); height, from the highest part of the beetle to elytral outer margins (TH); head width in frontal view, widest part (HW); pronotal length, from the middle of anterior margin to the base of pronotum (PL); pronotal width at widest part (PW); elytral length, along the suture, from the apex to the base including the scutellum (EL). Male and female genitalia were dissected, cleared in a 10% solution of NaOH by boiling for several minutes, and examined with an Olympus BX51 compound microscope.

Specimens were photographed with digital cameras (AxioCam HRc and Coolsnap–Pro *cf* & CRI Micro*Color), connected to the dissecting microscope. The software AxioVision Rel. 4.8 and Image–Pro Plus 5.1 were used to capture images from both cameras, and photos were cleaned up and laid out in plates with Adobe Photoshop CS 8.0.

Terminology follows Ślipiński (2007). Type specimens designated in the present paper are deposited at the Department of Entomology, South China Agriculture University (SCAU), Guangzhou, China.

## Taxonomy

### Genus Microserangium

Miyatake, 1961

http://species-id.net/wiki/Microserangium

Microserangium Miyatake, 1961a: 37. Type species, original designation, *Microserangium shikokense* Miyatake, 1961a.
Serangiella Chapin, 1940: 271. Unavailable name.

#### Diagnosis.

This genus is very similar to *Pangia* Wang & Ren, 2012 but it can be distinguished from the latter as follows: mandible reduced, apical tooth erect (Fig. 4), penis guide usually simple (Figs 25, 33, 41), ovipositor elongate-oval and without styli, spermatheca composed of two or three globular parts (Figs 10–11). In *Pangia*, the mandible is normal, apical tooth is bent, penis guide is strongly asymmetrical and complex, ovipositor is triangularly elongate and usually bearing short styli, and spermatheca has a large part and a small process.

This genus is also similar to *Catanella* Miyatake, 1961, but it can be distinguished from the latter as follows: mandible reduced with erect apical tooth (Fig. 4), antenna with 9 antennomeres, with antennomere 3 strongly triangular (Fig. 5). In *Catanella*, the mandible is normal, apical tooth is bent, antennae with 8 antennomeres, with antennomere 3 elongate and not expanded.

**Figures 1–11.**
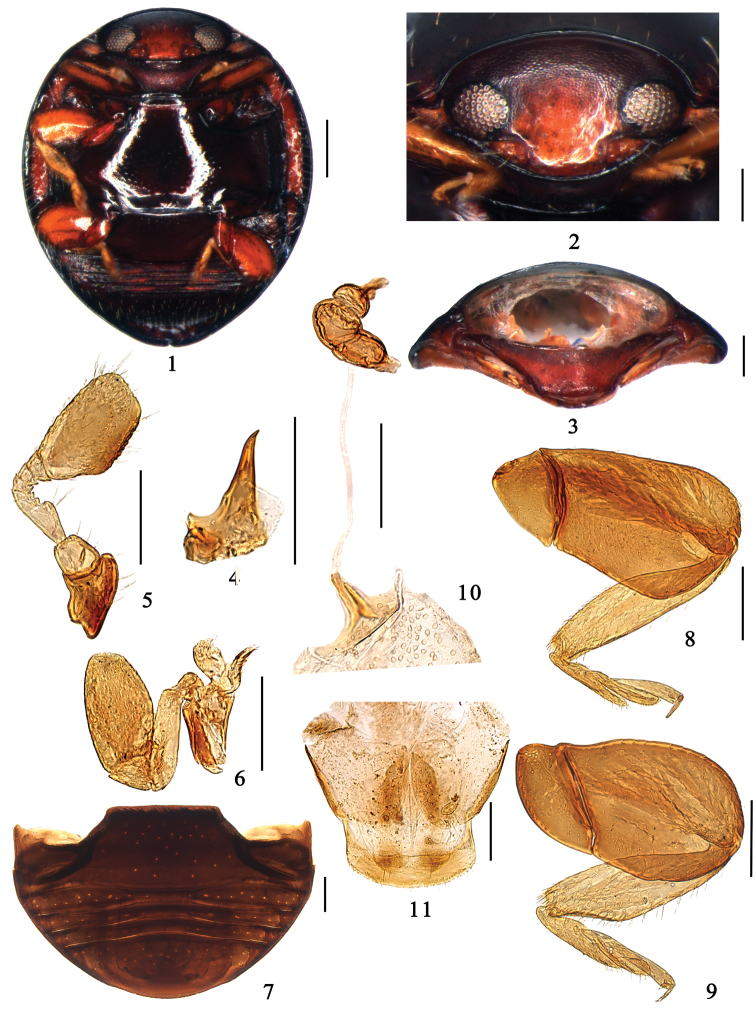
*Microserangium dactylicum* Wang & Ren, sp. n. **1** ventral view **2** head frontal view **3** prothorax **4** mandible **5** antenna **6** maxilla **7** abdomen **8** fore leg **9** hind leg **10–11** female genitalia. Scale bars: 0.1mm.

#### Description.

**Figures 12–20.**
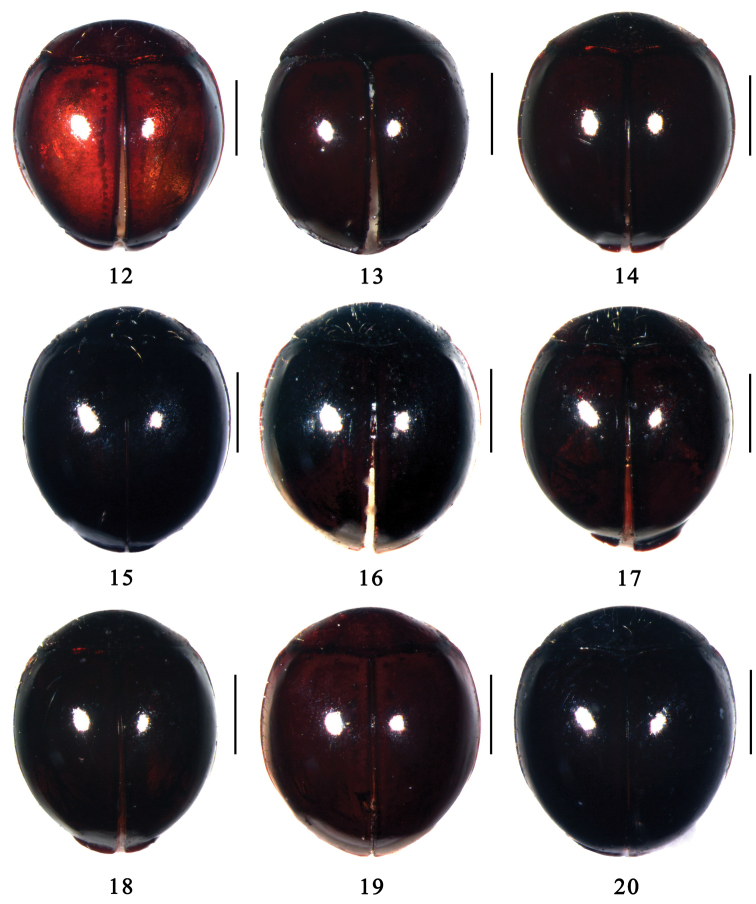
Dorsal view. **12** *Microserangium erythrinum* Wang & Ren, sp. n. **13** *Microserangium fuscum* Wang & Ren, sp. n. **14** *Microserangium glossoides* Wang & Ren, sp. n. **15** *Microserangium shennongensis* Wang & Ren, sp. n. **16** *Microserangium semilunatum* Wang & Ren, sp. n. **17** *Microserangium hainanensis* (Miyatake, 1961) **18** *Microserangium deltoides* Wang & Ren, sp. n. **19** *Microserangium okinawense* (Miyatake, 1961) **20** *Microserangium dactylicum* Wang & Ren, sp. n. Scale bars: 0.2mm.

Body minute, hemispherical with head in repose drawn into prothorax and closely fitting ventrally against prominent prosternal lobe (Fig. 1); dorsum glabrous, pronotum and elytral outer margins with sparse long setation (Figs 12–20). Head transverse, ventrally flattened with clypeal region prominent anteriorly (Fig. 2); frontoclypeus deeply emarginated around exposed antennal insertions. Mandible reduced, apical tooth erect (Fig. 4). Antenna with 9 antennomeres; antennomere 1 stout, antennomere 2 globular and smaller than 1, antennomere 3 strongly triangular; club, oval and flat with apex angulate (Fig. 5). Terminal maxillary palpomere always longer than wide, barrel-shaped, truncate at apex (Fig. 6).

Pronotum strongly transverse, anterior corner rounded. Scutellum relatively large, triangular. Elytra usually smooth without visible punctures. Wings with greatly reduced venation. Prosternum strongly prominent medially forming a broad lobe concealing mouthparts from below; prosternal process subtruncate apically, broad, without carinae (Fig. 3). Mesoventrite very short and broad. Metaventrite large and broad, surface shining and glabrous (Fig. 1). Epipleuron moderately narrow, incomplete, reaching 2/3 of elytral length, with clearly delimited cavities to accommodate apices of meso- and metafemora. Abdomen with 5 ventrites (Fig. 7), ventrite 1 and 5 much longer than 2–4; hind margin of terminal ventrite rounded and smooth. Abdominal postcoxal lines incomplete, reaching lateral margin of ventrite, without associated pits or pores. Femora, especially profemur, broad, flat, closely fitting into depressions on ventral surface, protecting tibiae and tarsi from below; meso- and metatibiae conspicuously protuberant externally beyond middle, usually triangular; tarsus with 3 (Figs 8–9), rarely 4 tarsomeres.

Male genitalia: tegmen strongly asymmetrical, parameres extremely short or distinctly reduced sparsely setose apically (Figs 24–25). Female genitalia: ovipositor oval, without styli; spermatheca small and well sclerotised (Figs 10–11).

**Figures 21–36.**
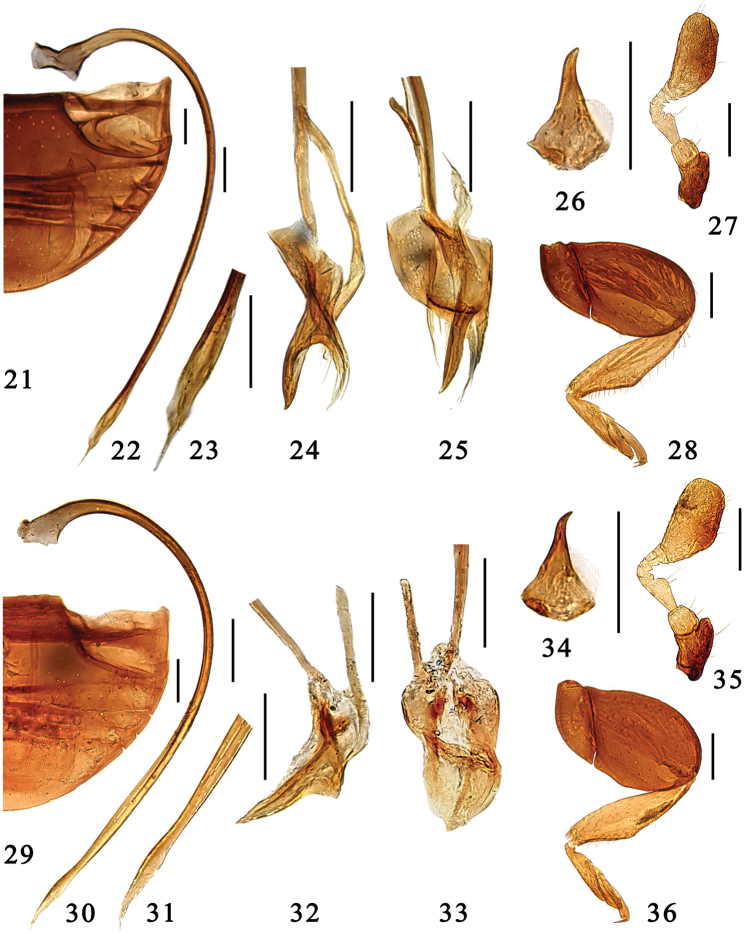
**21–28** *Microserangium erythrinum* Wang & Ren, sp. n. **21** abdomen **22–25** male genitalia: **22** penis **23** apex of penis **24** tegmen, lateral view **25** tegmen, ventral view **26** mandible **27** antenna **28** hind leg **29–36** *Microserangium fuscum* Wang & Ren, sp. n. **29** abdomen **30–33** male genitalia: **30** penis **31** apex of penis **32** tegmen, lateral view **33** tegmen, ventral view **34** mandible **35** antenna **36** hind leg. Scale bars: 0.1mm.

#### Distribution.

China, India, Japan, Mayotte Island (Indian Ocean), Sri Lanka, Vietnam.

#### Key to species of *Microserangium* from China

**Table:** 

1	Elytra uniformly black	2
–	Elytral disk yellow or burgundy with dark brown margins (Fig. 12). TL: 1.32–1.42 mm, TW: 1.20–1.30 mm	*Microserangium erythrinum*
2	Frons densely punctate, with variable, medium to large punctures	3
–	Frons finely, sparsely punctate; punctures of one size, without large ones	6
3	Pronotum densely covered with large and fine punctures. Frons with medium to large punctures	4
–	Pronotum sparsely covered with fine punctures. Frons with four to six moderately large punctures and many fine punctures. TL: 1.15–1.29 mm, TW: 1.02–1.15mm	*Microserangium fuscum*
4	Parameres short and inconspicuous	5
–	Parameres relatively long and conspicuous (Fig. 40). TL: 1.38–1.62mm, TW: 1.19–1.48mm	*Microserangium glossoides*
5	Penis guide in ventral view slender and moderately curved, apex pointed (Fig. 48). TL: 1.25–1.35mm, TW: 1.09–1.15 mm	*Microserangium shennongensis*
–	Penis guide in ventral view small and semilunate (Fig. 57). TL: 1.42–1.55mm, TW: 1.24–1.35mm	*Microserangium semilunatum*
6	Punctures at centre of metaventrite moderately large and densely distributed. [TL: 1.52–1.62 mm, TW: 1.35–1.45mm]	*Microserangium hainanensis*
–	Punctures on metaventrite fine and sparsely distributed	7
7	Pronotum black	8
–	Anterior corners of pronotum yellowish brown. [TL: 1.19–1.35mm, TW: 1.02–1.15mm]	*Microserangium deltoides*
8	Inner arm of penis capsule small but conspicuous (Fig. 78). Apical half of the penis guide in ventral view flat and triangular, outer margin arcuate, apex slightly blunt; basal half as Fig. 81. TL: 1.35–1.42mm, TW: 1.15–1.25mm	*Microserangium okinawense*
–	Inner arm of penis capsule inconspicuous (Fig. 86). Apical half of the penis guide in ventral view flat and shovel-shaped, outer margin relatively straight, apex finger-shaped. Basal half as Fig. 89. TL: 1.25–1.38mm, TW: 1.12–1.22 mm	*Microserangium dactylicum*

### Microserangium erythrinum

Wang & Ren sp. n.

http://zoobank.org/86B9A892-300A-4B85-AF4F-B69F1E6A6943

http://species-id.net/wiki/Microserangium_erythrinum

Figures 12 , 21–28 , 93

#### Diagnosis.

This species can be distinguished from other *Microserangium* species by its yellow or burgundy elytral disk with dark brown margins and its penis guide acutely triangular (Figs 12, 25).

#### Description.

TL: 1.32–1.42mm, TW: 1.20–1.30mm, TH: 0.76–0.86mm, TL/TW: 1.09–1.10; PL/PW: 0.38–0.40; EL/EW: 0.88–0.89; HW/TW: 0.46; PW/TW: 0.73.

Body shiny and glabrous (Fig. 12). Head brown, frons yellowish brown. Pronotum brown to black, scutellum dark brown. Elytra with disk yellow to burgundy and margins dark brown. Underside dark red. Legs yellowish brown, tibiae and tarsi yellow.

Head transverse and ventrally flattened; frontal punctures large and sparsely distributed, with short sparsely distributed setae on frons; eyes round, large and coarsely faceted, widest interocular distance 0.39× width of head. Antennal club oval and flat, apex rounded (Fig. 27).

Pronotum transverse, anterior corners rounded. Pronotal disk with large and fine punctures both associated with long sparsely distributed setae, the large punctures distinctly larger than those on frons, 0.5–3.0 diameters apart. Elytra smooth and shiny with sparsely distributed long setae along margins, punctures inconspicuous. Prosternum mat and impunctate. Mesoventrite transverse, very short, surface mat, weakly furrowed. Metaventrite shiny and glabrous, punctures fine and sparsely distributed, 1.0–2.0 diameters apart. Meso- and metatibiae angulate externally beyond middle, almost triangular; tarsus with 3 tarsomeres (Fig. 28).

Male genitalia. Penis strongly curved along entire length, apex narrowed and acicular, penis capsule with short outer arm and indistinct inner one (Figs 22–23). Tegmen slender and strongly asymmetrical. Penis guide in lateral view slender, almost straight, pointed apically (Fig. 24); in ventral view acutely triangular (Fig. 25). Parameres distinct, small, about half as long as penis guide.

#### Types.

**Holotype** ♂: **China, Yunnan:** Menglun, Xishuangbanna, 21°55.27'N, 101°16.64'E, ca 550m, 21.viii.2005, Wang XM leg. **Paratypes (8)**: **Yunnan:** 1♀, Mengxing, Mengla, Xishuangbanna, 21°52.63'N, 101°27.07'E, ca 690m, 3.v.2008, Wang XM leg.; 1♀, Xishuangbanna Plant Park, Xishuangbanna, 21°56.05'N, 101°15.55'E, ca 550m, 22.viii.2005, Wang XM leg.; 1♀, Nuozadu, Simao, 22°34.0'N, 100°33.39'E, ca 750m, 12.v.2008, Wang XM leg.; 2♂♂, Dadugang, Puer, 22°22.35'N, 100°56.68'E, ca 950m, 5.v.2009, Ren SX & Wang XM leg.; 1♂1♀, Yaoqu, Mengla, 21°46.98'N, 101°29.34'E, ca 700m, 7.v.2009, Wang XM leg.; 1♂, Daheishan, Jiangcheng, 22°33.74'N, 101°50.87'E, ca 1300m, 17.v.2009, Wang XM leg.

#### Distribution.

China (Yunnan).

#### Etymology.

The specific epithet is formed from the Latin adjective *erythrinus* red colored, referring to the elytral disk being of yellow to burgundy color.

### Microserangium fuscum

Wang & Ren sp. n.

http://zoobank.org/1E69C318-0C83-4C22-A24A-870862CD263C

http://species-id.net/wiki/Microserangium_fuscum

Figures 13 , 29–36 , 93

#### Diagnosis.

This species can be distinguished from other *Microserangium* species by its relatively small body, dark brown dorsum (Fig. 13), fine and sparse pronotal punctation, rather small penis capsule (Fig. 30), and rather wide penis guide (Fig. 33).

#### Description.

TL: 1.15–1.29mm, TW: 1.02–1.15mm, TH: 0.63–0.69mm, TL/TW: 1.11–1.13; PL/PW: 0.34–0.44; EL/EW: 0.89–0.90; HW/TW: 0.45; PW/TW: 0.77.

Body shiny and glabrous (Fig. 13). Dorsum uniformly dark brown. Head brown. Underside reddish brown, legs yellowish brown, tibiae and tarsi yellow.

Head transverse and ventrally flattened; frons with four to six medium punctures and many fine punctures, and several long widely separated setae; eyes round, moderately large and coarsely faceted, widest interocular distance 0.57× width of head. Antennal club oval and flat, apex blunt (Fig. 35).

Pronotum transverse, anterior corners inconspicuous and blunt, glabrous, punctures fine and sparsely distributed, associated with long sparsely distributed setae. Elytra smooth and shiny, with sparse row of long setae along margins, punctures extremely fine and inconspicuous. Prosternum mat and impunctate. Mesoventrite transverse, very short, surface mat, weakly furrowed. Metaventrite shiny and glabrous, punctures in center fine, 1.0–3.0 diameters apart. Meso- and metatibiae angulate externally beyond middle, almost triangular; tarsus with 3 tarsomeres (Fig. 36).

Male genitalia. Penis strongly curved along entire length, apex narrowed and pointed, penis capsule small (Figs 30–31). Tegmen slender and strongly asymmetrical. Penis guide in lateral view slender, almost straight, apex pointed (Fig. 32), in ventral view flat and rather wide (Fig. 33). Parameres small and short, less than 1/3 length of penis guide.

#### Types.

**Holotype** ♂: **China, Hainan:** Wushi, 19°8.99'N, 109°53.84'E, ca 320m, 14.vii.1999, Peng ZQ leg.; **Paratypes (2)**: 1♀, same data as holotype; 1♂, Nanbin, 18°21.37'N, 109°11.0'E, ca 10m, 24.iii.1998, Peng ZQleg.

#### Distribution.

China (Hainan).

#### Etymology.

The specific epithet is formed from the Latin adjective *fuscus*, referring to the elytral disk being uniformly dark brown.

### Microserangium glossoides

Wang & Ren sp. n.

http://zoobank.org/829EA7BA-0BB7-4CDC-803E-A1F33A41A387

http://species-id.net/wiki/Microserangium_glossoides

Figures 14 , 37–44 , 93

#### Diagnosis.

This species can be distinguished from other *Microserangium* species by its male genitalia with relatively long parameres and tongue-shaped penis guide (Figs 40–41).

**Figures 37–52.**
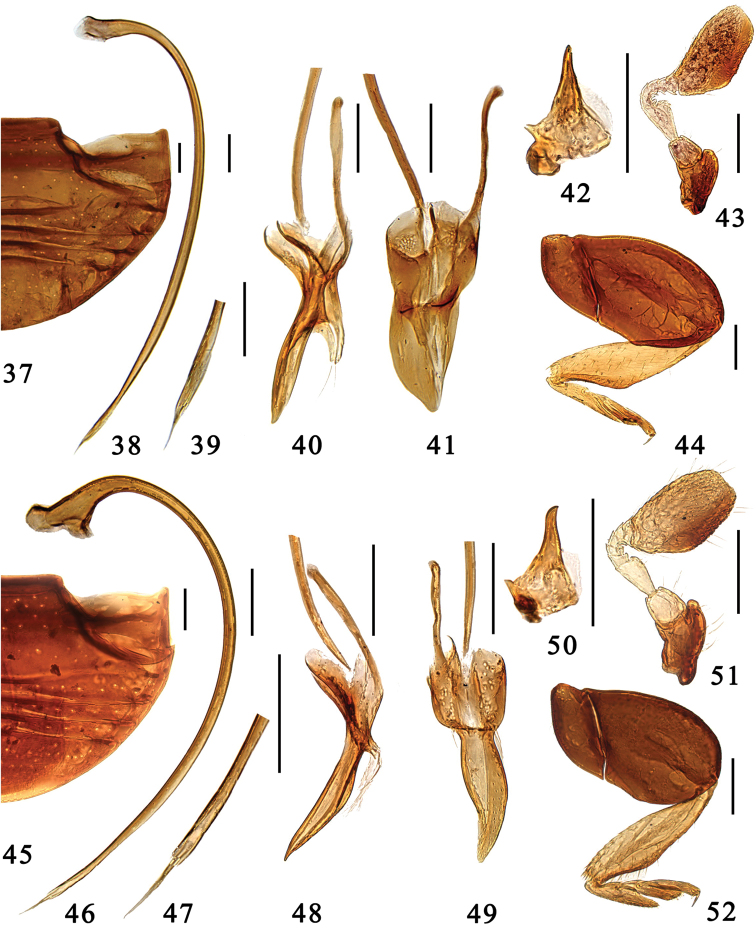
**37–44** *Microserangium glossoides* Wang & Ren, sp. n. **37** abdomen **38–41** male genitalia: **38** penis **39** apex of penis **40** tegmen, lateral view **41** tegmen, ventral view **42** mandible **43** antenna **44** hind leg **45–52** *Microserangium shennongensis* Wang & Ren, sp. n. **45** abdomen **46–49** male genitalia: **46** penis **47** apex of penis **48** tegmen, lateral view **49** tegmen, ventral view **50** mandible **51** antenna **52** hind leg. Scale bars: 0.1mm.

#### Description.

TL: 1.38–1.62mm, TW: 1.19–1.48mm, TH: 0.76–0.82mm, TL/TW: 1.09–1.17; PL/PW: 0.42–0.46; EL/EW: 0.89–1.00; HW/TW: 0.42; PW/TW: 0.73.

Body shiny and glabrous (Fig. 14). Dorsum uniformly dark brown to black. Head yellowish brown. Underside reddish brown, legs yellowish brown, tibiae and tarsi yellow.

Head transverse and ventrally flattened; frontal punctures medium-sized and densely distributed, 1.0–1.5 diameter apart, associated with several long sparsely distributed setae; eyes round, large and coarsely faceted, widest interocular distance 0.42× width of head. Antennal club oval and flat, apex angular (Fig. 43).

Pronotum transverse, anterior corners inconspicuous and blunt, Pronotal disk densley covered with large and fine punctures both associated with long sparsely distributed setae. Elytra smooth and shiny, with sparse row of long setae along margin, punctures extremely fine and inconspicuous. Prosternum mat and impunctate. Mesoventrite transverse, very short, surface mat, weakly furrowed. Metaventrite shiny and glabrous, punctures at center fine but conspicuous, 2.0–4.0 diameters apart. Meso- and metatibiae angulate externally beyond middle, almost triangular; tarsus with 3 tarsomeres (Fig. 44).

Male genitalia. Penis moderately curved, apex strongly narrowed and acicular, penis capsule with short outer arm and inconspicuous inner one (Figs 38–39). Tegmen slender and asymmetrical. Penis guide in lateral view slender, almost straight, apex pointed (Fig. 40), in ventral view flat and tongue-shape, apex slightly pointed (Fig. 41). Parameres moderately long, slightly less than ½ length of penis guide.

#### Types.

**Holotype** ♂: **China, Hainan:** Bawangling, 19°05.65'N, 109°6.73'E, ca 330m, 21.iii.1996, Peng ZQ leg. **Paratypes (11)**: **Hainan:** 5♂♂, same data to holotype; 2♂♂, Wuzhishan, 18°47.0'N, 109°31.98'E, ca 650m, viii.1995, Peng ZQleg.; 1♂, Limushan, 19°16.08'N, 109°47.32'E, ca 280m, 21.iv. 1996, Peng ZQleg.; 1♂, Shijing, Diaoluoshan, 18°56.15'N, 109°56.90'E, ca 200m, ix. 1995, Peng ZQleg.; 1♀, Wuzhishan, 18°47'N, 109°31.98'E, ca 650m, 22.xi.1991, Peng ZQ leg.; 1♂, Limushan, 19°16.08'N, 109°47.32'E, ca 280m, 22.vii.2006, Wang XMleg.

#### Distribution.

China (Hainan).

#### Etymology.

The specific epithet is formed from the Latin adjective *glossoides*, referring to the tongue-shaped penis guide.

### Microserangium shennongensis

Wang & Ren sp. n.

http://zoobank.org/2E6728C1-68D9-4425-B824-A0FBB2FC991B

http://species-id.net/wiki/Microserangium_shennongensis

Figures 15 , 45–52 , 93

#### Diagnosis.

This species is similar to *Microserangium glossoides*, from which it differs in having a relatively large outer arm of the penis capsule, small parameres, and narrow and curved penis guide (Figs 46–49). In *Microserangium glossoides*, the penis capsule is inconspicuous, the parameres are distinctly longer than in *Microserangium shennongensis*, and the penis guide is tongue-shaped (Figs 38–41).

#### Description.

TL: 1.25–1.35mm, TW: 1.09–1.15mm, TH: 0.66–0.69mm, TL/TW: 1.15–1.17; PL/PW: 0.42–0.44; EL/EW: 0.94–1.11; HW/TW: 0.42; PW/TW: 0.73.

Body shiny and glabrous (Fig. 15). Dorsum uniformly black. Head reddish brown. Underside dark brown, legs brown, tibiae and tarsi yellow.

Head transverse and ventrally flattened; frontal punctures large, conspicuous and densely distributed, 0.5–1.0 diameter apart, associated with long sparsely distributed setae; eyes round, large and coarsely faceted, widest interocular distance 0.50× width of head. Antennal club oval and flat, apex truncated (Fig. 51).

Pronotum short and strongly transverse, anterior corners inconspicuous and blunt. Pronotal disk with densely distributed large and fine punctures both associated with long sparsely distributed setae. Elytra smooth and shiny, with sparse row of long setae along margins, punctures extremely fine and inconspicuous. Prosternum mat and impunctate. Mesoventrite transverse, very short, surface mat, weakly furrowed. Metaventrite shiny and glabrous, punctures fine and densely distributed, 1.5–2.0 diameters apart. Meso- and metatibiae weakly angulate externally beyond middle; tarsus with 3 tarsomeres (Fig. 52).

Male genitalia. Penis strongly curved along entire length, apex narrowed and acicular, penis capsule with short but distinct outer arm and small inner one (Figs 46–47). Tegmen rather slender and strongly asymmetrical. Penis guide in lateral view slender, outer margin arcuate, apex pointed (Fig. 48), in ventral view slender and moderately curved, apex pointed (Fig. 49). Parameres inconspicuous, small.

#### Types.

**Holotype** ♂: **China, Hunan:** Shennong Valley National Forest Park, Yanling, 26°29.95'N, 114°0.18.98'E, ca 800m, 9.x.2010, Wang XM leg. **Paratypes (4)**: **Hunan:** 1♂3♀♀, same data as the holotype.

#### Distribution.

China (Hunan).

#### Etymology.

The specific epithet refers to the Shennong Valley National Forest Park, the type locality of this ladybird.

### Microserangium semilunatum

Wang & Ren sp. n.

http://zoobank.org/0B55E091-1323-469F-A479-57E7F47388D4

http://species-id.net/wiki/Microserangium_semilunatum

Figures 16 , 53–60 , 93

#### Diagnosis.

The male genitalia of this species are similar to *Microserangium erythrinum*, but this species can be distinguished from the latter by its uniformly dark elytra and semilunate penis guide (Figs 16, 56–57). In *Microserangium erythrinum*,theelytral disk is yellow or burgundy with dark brown margins and the penis guide is acutely triangular (Figs 12, 24–25).

**Figures 53–68.**
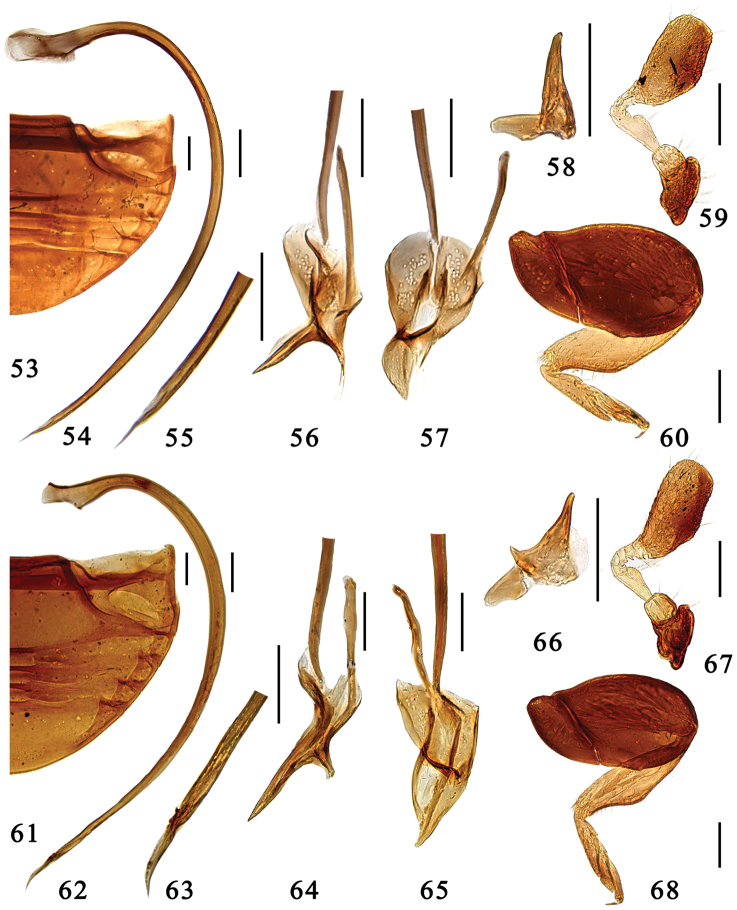
**53–60** *Microserangium semilunatum* Wang & Ren, sp. n. **53** abdomen **54–57** male genitalia: **54** penis **55** apex of penis **56** tegmen, lateral view **57** tegmen, ventral view **58** mandible **59** antenna **60** hind leg **61–68** *Microserangium hainanensis* Miyatake, 1961 **61** abdomen **62–65** male genitalia: **62** penis **63** apex of penis **64** tegmen, lateral view **65** tegmen, ventral view **66** mandible **67** antenna **68** hind leg. Scale bars: 0.1mm.

#### Description.

TL: 1.42–1.55mm, TW: 1.24–1.35mm, TH: 0.76–0.86mm, TL/TW: 1.14–1.15; PL/PW: 0.40–0.44; EL/EW: 0.93–0.98; HW/TW: 0.44; PW/TW: 0.73.

Body shiny and glabrous (Fig. 16). Dorsum uniformly black. Head black, clypeus brown. Underside black, legs dark brown, tibiae and tarsi yellow.

Head transverse and ventrally flattened; frontal punctures large and densely distributed, 0.3–1.0 diameter apart, associated with long sparsely distributed setae; eyes round, large and coarsely faceted, widest interocular distance 0.50× width of head. Antennal club oval and flat, apex blunt (Fig. 59).

Pronotum short and strongly transverse, anterior corner inconspicuous and blunt, mat and shagreened. Pronotal disk with densely distributed large and fine shallow punctures both associated with long sparsely distributed setae. Elytra smooth and shiny, with sparse row of long setae along margins, punctures extremely fine and inconspicuous. Prosternum mat and impunctate. Mesoventrite transverse, very short, surface mat weakly furrowed. Metaventrite shiny and glabrous, punctures fine and sparsely distributed, 2.0–3.0 diameters apart. Meso- and metatibiae strongly angulate externally beyond middle; tarsus with 3 tarsomeres (Fig. 60).

Male genitalia. Penis strongly curved along entire length, apex narrowed and acicular, penis capsule with large outer arm and inconspicuous inner one (Figs 54–55). Tegmen rather slender and strongly asymmetrical. Penis guide in lateral view small, almost straight, apex pointed (Fig. 56), in ventral view small and semilunate (Fig. 57). Parameres conspicuous.

#### Types.

**Holotype** ♂: **China, Hainan:** Bawangling, 19°05.65'N, 109°6.73'E, ca 330m, 21.iii.1996, Peng ZQ leg. **Paratypes** (12): **Hainan:** 4♂♂1♀, same data to holotype; 1♂1♀, Bawangling, 19°05.65'N, 109°6.73'E, ca 330m, 3.ix.1998, Peng ZQleg.; 1♂, Bawangling, 19°05.65'N, 109°6.73'E, ca 330m, 20.iv.2000, Peng ZQleg.; 1♂1♀, Diaoluoshan, 18°56.15'N, 109°56.90'E, ca 200m, 26.vii.2006, Wang XM leg.; 1♂, Diaoluoshan, 18°56.15'N, 109°56.90'E, ca 200m, 7.v.2005, Wang XM leg.

#### Distribution.

China (Hainan).

#### Etymology.

The specific epithet is formed from the the Latin adjective *semilunatus*, referring to the semilunate penis guide.

### Microserangium hainanensis

Miyatake, 1961

http://species-id.net/wiki/Microserangium_hainanensis

Figures 17 , 61–68 , 93

Microserangium hainanensis Miyatake, 1961b: 144.
Serangiella hainanensis : Ślipiński and Burckhardt 2006: 50.

#### Diagnosis.

This species can be distinguished as follows: frontal punctures fine and sparsely distributed, pronotum densely covered with large punctures associated with long sparsely distributed setae, and punctures in central part of metaventrite moderately large and densely distributed. The penis guide of the male genitalia is also unique (Fig. 65).

#### Description.

TL: 1.52–1.62mm, TW: 1.35–1.45mm, TH: 0.86–0.89mm, TL/TW: 1.11–1.12; PL/PW: 0.44–0.45; EL/EW: 0.90–0.91; HW/TW: 0.43; PW/TW: 0.70.

Body shiny and glabrous (Fig. 17). Dorsum uniformly black. Head dark brown, frons brown. Underside dark brown, legs reddish brown, tibiae and tarsi yellow.

Head transverse and ventrally flattened; frontal punctures fine and sparsely distributed, 1.0–2.0 diameters apart, associated with long sparsely distriuted setae, eyes round, large and coarsely faceted, widest interocular distance 0.47× width of head. Antennal club oval and flat, apex blunt (Fig. 67).

Pronotum strongly transverse, anterior corners inconspicuous and blunt, mat and shagreened. Pronotal disk with densely distributed large punctures associated with long sparsely distributed setae. Elytra smooth and shiny, with long sparsely distributed setae along margins, punctures extremely fine and inconspicuous. Prosternum mat and impunctate. Mesoventrite transverse, very short, surface mat weakly furrowed. Metaventrite shiny and glabrous, with densely distributed medium size punctures at center, 1.0–2.0 diameters apart. Meso- and metatibiae angulate externally beyond middle; tarsus with 3 tarsomeres (Fig. 68).

Male genitalia. Penis strongly curved, apex narrowed and pointed, penis capsule with conspicuous outer arm (Figs 62–63). Tegmen slender and strongly asymmetrical. Penis guide in lateral view slender, straight, apex pointed (Fig. 64), in ventral view acute triangular, apex pointed (Fig. 65). Parameres conspicuous, small, short, rectangular with penis guide (Fig. 64).

#### Specimens examined.

**China, Hainan:** 2♂♂, Tianchi, Jingfengling, 18°44.42'N, 108°51.80'E, ca 820m, viii.1995, Peng ZQ leg. 2♂♂, Xinan, Diaoluoshan, 18°56.15'N, 109°56.90'E, 18.ix.1995, Peng ZQ leg.; 1♂, Bawangling, 19°05.65'N, 109°6.73'E, ca 330m, 5.v.2005, Wang XM leg.

#### Distribution.

China (Hainan).

### Microserangium deltoides

Wang & Ren sp. n.

http://zoobank.org/ABCE9651-A3E7-4ECF-8BB7-2DFA0C4E7056

http://species-id.net/wiki/Microserangium_deltoides

Figures 18 , 69–76 , 93

#### Diagnosis.

This species is similar to *Microserangium okinawense*, but can be separated from the latter by the inconspicuous inner arm of the penis capsule and triangular penis guide (Figs 70–73). In *Microserangium okinawense*, the inner arm of the penis capsule is small but distinct, and the penis guide is almost triangular with inner and outer margins arcuate (Figs 78–81).

**Figures 69–84.**
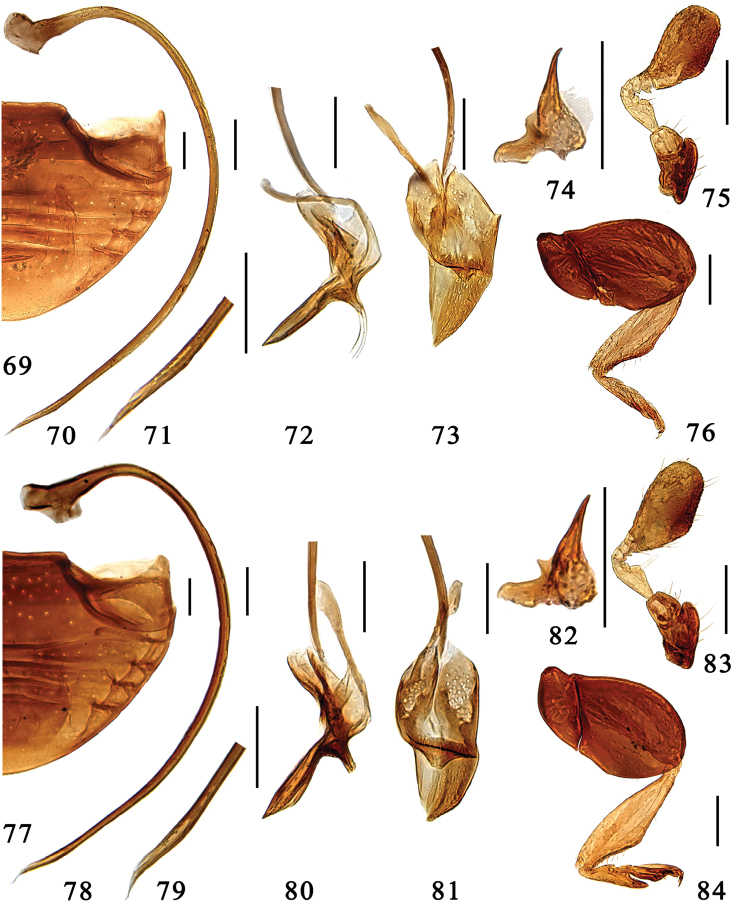
**69–76** *Microserangium deltoides* Wang & Ren, sp. n. **69** abdomen **70–73** male genitalia: **70** penis **71** apex of penis **72** tegmen, lateral view **73** tegmen, ventral view **74** mandible **75** antenna **76** hind leg **77–84** *Microserangium okinawense* Miyatake, 1961 **77** abdomen **78–81** male genitalia: **78** penis **79** apex of penis **80** tegmen, lateral view **81** tegmen, ventral view **82** mandible **83** antenna **84** hind leg. Scale bars: 0.1mm.

#### Description.

TL: 1.19–1.35mm, TW: 1.02–1.15mm, TH: 0.66–0.76mm, TL/TW: 1.16–1.17; PL/PW: 0.38–0.44; EL/EW: 0.87–1.00; HW/TW: 0.43; PW/TW: 0.71.

Body shiny and glabrous (Fig. 18). Dorsum uniformly black, anterior corner of pronotum yellowish brown. Head dark brown, except frons medium brown. Underside dark brown, legs yellowish brown, tibiae and tarsi yellow.

Head transverse and ventrally flattened; frontal punctures fine and sparsely distributed, 1.0–2.0 diameters apart, with long sparsely distributed setae; eyes round, large and coarsely faceted, widest interocular distance 0.40× width of head. Antennal club oval and flat, apex blunt (Fig. 75).

Pronotum strongly transverse, anterior corners inconspicuous and blunt, mat and shagreened. Pronotal disk with densely distributed large and fine punctures both associated with long sparsely distributed setae, the finer punctures slightly deeper than the large ones. Elytra smooth and shiny, with long sparsely distributed setae along margins, punctures extremely fine and inconspicuous. Prosternum mat and impunctate. Mesoventrite transverse, very short, surface mat, weakly furrowed. Metaventrite shiny and glabrous, punctures fine and sparsely distributed, 1.5–3.0 diameters apart. Meso- and metatibiae distinctly angulate externally beyond middle, almost triangular; tarsus with 3 tarsomeres (Fig. 76).

Male genitalia. Penis strongly curved along entire length, apex narrowed and pointed, penis capsule with large outer arm and inconspicuous inner one (Figs 70–71). Tegmen slender and asymmetrical. Penis guide in lateral view slender and straight, apex pointed (Fig. 72), in ventral view flat and triangular, apex pointed (Fig. 73). Parameres conspicuous, small (Fig. 72).

#### Types.

**Holotype** ♂: **China, Guangxi:** Pinglongshan, Fulong, Shangsi, 21°49.88'N, 107°56.79'E, ca 160m, 29.vii.2005, Wang XM leg. **Paratypes (28): Guangxi:** 1♂4♀♀, same data to holotype; 6♂♂3♀♀, Fulong, Shiwandashan, Shangsi, 21°49.88'N, 107°56.79'E, ca 160m, 7.xi.2004, Wang XM et al.leg.; 2♂♂11♀♀, Hongqilinchang, Shiwandashan, Shangsi, 21°52.79'N, 107°55.05'E, ca 900m, 7.xi.2004, Wang XM et al.leg.; **Guizhou:** 1♂, Xianheping, Anlong, 24°58.66'N, 105°36.45'E, ca 1500m, 12.ix.2007, Wang XM leg.

#### Distribution.

China (Guangxi, Guizhou).

#### Etymology.

The specific epithet is formed from the Latin adjective *deltoides*, referring to the triangular penis guide.

### Microserangium okinawense

Miyatake, 1961

http://species-id.net/wiki/Microserangium_okinawense

Figures 19 , 77–84 , 93

Microserangium okinawense Miyatake, 1961a: 131; Sasaji 1971: 64.
Serangiella okinawense : Ślipiński and Burckhardt 2006: 50; Ren et al. 2009: 38.

#### Diagnosis.

This species is close to *Microserangium deltoides*. The differences between these two are discussed in the diagnosis of *Microserangium deltoides*.

#### Description.

TL: 1.35–1.42mm, TW: 1.15–1.25mm, TH: 0.76–0.79mm, TL/TW: 1.13–1.17; PL/PW: 0.40–0.42; EL/EW: 0.95–1.00; HW/TW: 0.42; PW/TW: 0.68.

Body shiny and glabrous (Fig. 19). Dorsum uniformly dark brown to black. Head reddish brown. Underside reddish brown, legs yellowish brown, tibiae and tarsi yellow.

Head transverse and ventrally flattened; frontal punctures fine and inconspicuous, with long sparsely distributed setae; eyes round, large and coarsely faceted, widest interocular distance 0.44× width of head. Antennal club oval and flat, apex blunt (Fig. 83).

Pronotum short and strongly transverse, anterior corners inconspicuous and blunt, mat and shagreened. Pronotal disk with densely distributed large and fine shallow punctures both associated with long sparsely distributed setae. Elytra smooth and shiny, with sparse row of long setae along margins, punctures extremely fine and inconspicuous. Prosternum mat and impunctate. Mesoventrite transverse, very short, surface mat weakly furrowed. Metaventrite shiny and glabrous, punctures in center fine and conspicuous, 1.0–2.0 diameters apart. Meso- and metatibiae protuberant externally beyond middle, almost triangular; tarsus with 3 tarsomeres (Fig. 84).

Male genitalia. Penis strongly curved along entire length, apex narrowed and pointed, penis capsule conspicuous (Figs 78–79). Tegmen rather slender and strongly asymmetrical. Penis guide in lateral view slender, straight, widest in apical 1/3, narrowed at base, apex pointed (Fig. 80); in ventral view flat and shovel-shaped, apex slightly blunt (Fig. 81). Parameres conspicuous, small (Fig. 80).

#### Specimens examined.

**China, Taiwan:** 2♂♂1♀, Pingdong, Shuangliu, 22°13.07'N, 120°47.77'E, 200–400m, 21. X. 2012, S. Ren SX et al. leg.; 1♂1♀, Jiaxian & Tengzhi, 23°4.38'N, 120°36.94'E, 370m-1450m, 25. X. 2012, S. Ren SX et al. leg.; **Hainan:** 4♂♂, Wufenchang, Limushan, 19°16.23'N, 109°47.48'E, ca 280m, ix.1996, Peng ZQ leg. 2♂♂3♀♀, Bawangling, 19°05.65'N, 109°6.73'E, ca 330m, 5.v.2005, Wang XMleg.; 1♂, Yinggeling, 19°02.25'N, 109°33.85'E, ca 830m, 23.xi.1997, Peng ZQleg.; 1♀, Yinggeling, 19°02.25'N, 109°33.85'E, ca 830m, 8.v.2005, Wang XM leg.; 2♀♀, Limushan, 19°16.08'N, 109°47.32'E, ca 280m, 22.vii.2006, Wang XM leg.

#### Distribution.

China (Hainan, Taiwan).

### Microserangium dactylicum

Wang & Ren sp. n.

http://zoobank.org/AED888D3-8A64-4944-B27A-181BDF36DC3E

http://species-id.net/wiki/Microserangium_dactylicum

Figures 20 , 85 –93

#### Diagnosis.

This species is similar to *Microserangium bacthaiensis* Hoàng, 1978 in morphological characters and male genitalia, but it can be distinguished by the different shape of the penis guide.

#### Description.

TL: 1.25–1.38mm, TW: 1.12–1.22mm, TH: 0.76–0.82mm, TL/TW: 1.12–1.14; PL/PW: 0.42–0.44; EL/EW: 0.94–0.95; HW/TW: 0.43; PW/TW: 0.76.

Body shiny and glabrous (Fig. 20). Dorsum uniformly black. Head brown, except frons yellowish brown. Underside dark brown, except prosternum brown. Legs yellowish brown, tibiae and tarsi yellow (Fig. 1).

Head transverse and ventrally flattened; frontal punctures inconspicuous and sparsely distributed, with long sparsely distributed setae (Fig. 2); eyes round, large and coarsely faceted, widest interocular distance 0.44× width of head (Fig. 2). Antennal club, oval and flat, apex angular (Fig. 5).

Pronotum short and strongly transverse, with anterior corners inconspicuous and blunt, mat and shagreened. Pronotal disk with densely distributed large and fine punctures both associated with long sparsely distributed setae, punctures inconspicuous. Elytra smooth and shiny, with several long setae along margins, punctures extremely fine and inconspicuous. Prosternum mat and impunctate. Mesoventrite transverse, very short, surface mat, weakly furrowed (Fig. 3). Metaventrite shiny and glabrous, punctures fine and sparsely distributed, 3.0–4.0 diameters apart. Meso- and metatibiae protuberant externally beyond middle; tarsus with 3 tarsomeres (Fig. 9).

Male genitalia. Penis strongly curved along entire length, apex strongly narrowed and acicular, penis capsule with large outer arm and inconspicuous inner one (Figs 86–87). Tegmen rather slender and strongly asymmetrical. Penis guide in lateral view slender, straight, apex pointed (Fig. 88), in ventral view flat and triangular, apex finger-shaped (Fig. 89). Parameres conspicuous, small (Fig. 88).

**Figures 85–92.**
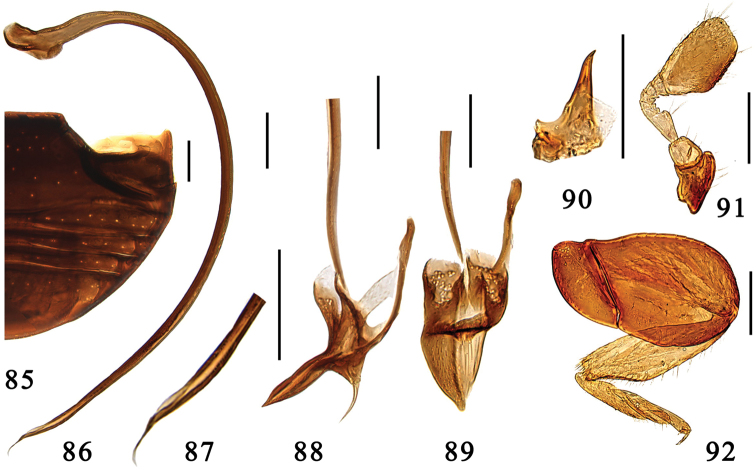
*Microserangium dactylicum* Wang & Ren, sp. n. **85** abdomen **86–89** male genitalia: **86** penis **87** apex of penis **88** tegmen, lateral view **89** tegmen, ventral view **90** mandible **91** antenna **92** hind leg. Scale bars: 0.1mm.

#### Types.

**Holotype** ♂: **China, Yunnan:** Dadugang, Puer, 22°22.35'N, 100°56.68'E, ca 950m, 26.iv.2008, Wang XM leg. **Paratypes** (20): **Yunnan:** 3♂♂2♀♀, same data to holotype; 2♂♂2♀♀, Tongbiguan, Nabang, Yingjiang, 24°37.86'N, 97°34.75'E, ca 1000m, 22–23.v.5.2008, Wang XM et al.leg.; 2♂♂5♀♀, Ganlongjing, Lianhuatan, Hekou, 22°56.59'N, 103°31.68'E, ca 710m, 20.v.2009, Wang XM et al. leg.; 1♀, Yaoqu, Mengla, 700m, 7.v.2009, Wang XM leg.; 1♂, Ainiguzhai, Jinghong, 21°54.67'N, 101°10.31'E, ca 660m, 11.v.2009, Wang XM leg.; 1♂, Lianhuatan, Hekou, 22°53.86'N, 103°34.04'E, ca 900m, 22.iv.2008, Wang XM leg.; 1♂, Daheishan, Jiangcheng, 22°33.62'N, 101°50.16'E, ca 1240m, 17.v.2009, Wang XM leg.

#### Distribution.

China (Yunnan).

**Figure 93.**
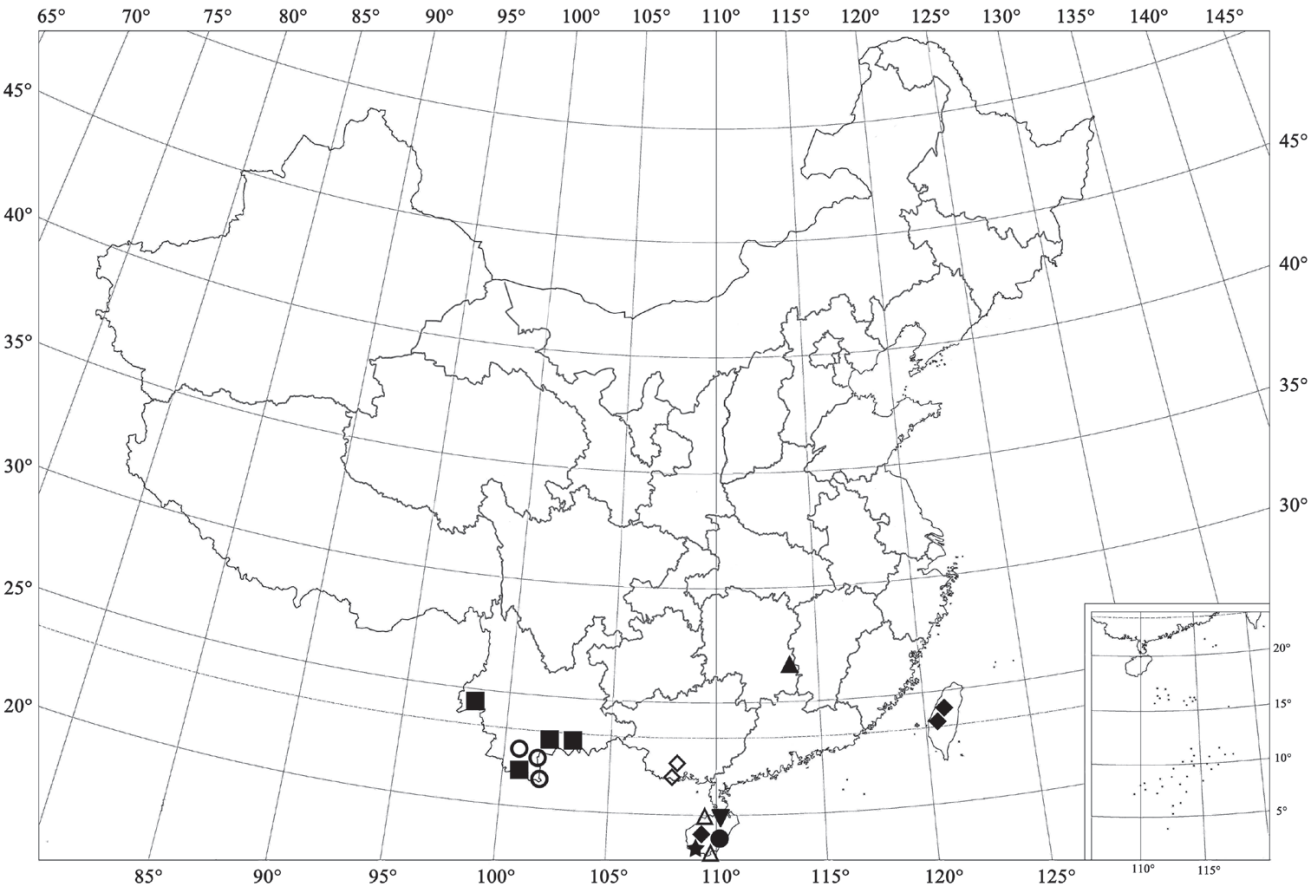
Distribution map. ○ *Microserangium erythrinum* Wang & Ren, sp. n. ● *Microserangium fuscum* Wang & Ren, sp. n. △ *Microserangium glossoides* Wang & Ren, sp. n. ▲ *Microserangium shennongensis* Wang & Ren, sp. n. ▼ *Microserangium semilunatum* Wang & Ren, sp. n. ★ *Microserangium hainanensis* Miyatake, 1961 ◇ *Microserangium deltoides* Wang & Ren, sp. n. ◆ *Microserangium okinawense* Miyatake, 1961 ■ *Microserangium dactylicum* Wang & Ren, sp. n.

#### Etymology.

The specific epithet is formed from the Latin adjective *dactylicus*, referring to the finger-shaped apex of the penis guide.

## Supplementary Material

XML Treatment for Microserangium

XML Treatment for Microserangium erythrinum

XML Treatment for Microserangium fuscum

XML Treatment for Microserangium glossoides

XML Treatment for Microserangium shennongensis

XML Treatment for Microserangium semilunatum

XML Treatment for Microserangium hainanensis

XML Treatment for Microserangium deltoides

XML Treatment for Microserangium okinawense

XML Treatment for Microserangium dactylicum
